# Interleukin-22 protects from endotoxemia by inducing suppressive F4/80^+^Ly6G^hi^Ly6C^hi^ cells population

**DOI:** 10.1186/s12865-022-00511-6

**Published:** 2022-09-19

**Authors:** Chang Yu, Qihua Ling, Junzhe Jiao, Juhong Liu, Zhihua Huang, Fang Wang, Xuehua Sun, Xiaoni Kong

**Affiliations:** 1 Central Laboratory, Department of Liver Diseases, Shuguang Hospital Affiliated to Shanghai University of Chinese Traditional Medicine, 528 Zhangheng Road, Shanghai, 201203 China; 2 Department of Emergency Internal Medicine, Shuguang Hospital Affiliated to Shanghai University of Chinese Traditional Medicine, Shanghai, China; 3 Evive Biotechnology (Shanghai) Ltd, Shanghai, China

**Keywords:** IL-22, Endotoxemia, Immunosuppressive macrophages, S100A9

## Abstract

**Background:**

Excessive inflammatory response is the primary cause of early death in patients with endotoxemia. Interleukin 22 (IL-22) has been shown to play critical roles in the modulation of infectious diseases, but its function in regulating immune responses during endotoxemia remains unclear.

**Methods:**

Lipopolysaccharide (LPS) was used to induce endotoxemia mouse model with or without a recombinant fusion protein containing human IL-22 (F-652). IL-6, TNF-α, IL-1β, and MCP-1 were measured by ELISA assays. The type of macrophage was assessed by flow cytometry. Real-time PCR was used to detect the expression of S100A9.

**Results:**

We found that F-652 injection significantly improved the survival rates and reduced pro-inflammatory cytokines (IL-6, TNF-a, IL-1β, MCP-1) in LPS-induced endotoxemia mice. However, the mice injected with F-652 had a higher number of infiltrated immune cells after LPS treatment, suggesting an impaired immune response. Flow cytometry analysis showed a higher number of F4/80^+^Ly6G^hi^Ly6C^hi^ cells that highly expressed M2-like macrophage markers (Ym1, Arg, CCL17) in the peritoneal cavity of the F-652-treated endotoxemia mice. Further investigation found that these suppressive M2 macrophages might be induced by F-652 since the F-652 treatment could increase S100A9 in vitro.

**Conclusions:**

Our study suggests that IL-22 has a protective role against endotoxemia by inducing the development of immunosuppressive cells through S100A9.

## Background

Endotoxemia remains a serious problem in hospitals, especially in ICUs. It has been considered as a severe clinical problem and a global public health threat. According to the epidemiology of endotoxemia in Chinese ICUs, endotoxemia affects 20% of ICU patients, its incidence in ICUs is about 20.6%, and the 90-day mortality rate is around 35.5% [1]. Since 2016, endotoxemia has been defined as life-threatening organ dysfunction caused by the dysregulated host response to infection [2]. Antibiotics are usually used as clinically effective drugs to treat endotoxemia, but with the emergence of drug-resistant bacteria, the therapeutic effect of antibiotics is greatly affected [3]. According to the organization for economic co-operation and development (OECD), if therapies are not improved, more than 2.4 million people will die of multi-resistant bacteria within the next 30 years in Europe, North America, and Australia [4]. Therefore, finding new diagnostic markers and treatments for endotoxemia is the key to reducing the mortality of endotoxemia.

In healthy conditions, the immune system is balanced, but this balance is disturbed in endotoxemia patients. The early death in endotoxemia patients is mainly due to host’s excessive inflammatory reaction to infection, and the late death in endotoxemia patients is mainly due to immune suppression. Since as early as the 1990s, many clinical studies have focused on limiting excessive inflammation for endotoxemia. However, there is still no substantial success [5]. Therefore, it is extremely important to suppress the excessive inflammatory response in the early stage of endotoxemia.

Interleukin-22 (IL-22) is a new cytokine discovered in 2000 and is classified as a member of the IL-10 family. IL-22 is mainly produced by activated T cells, NK cells, and innate lymphoid cells (ILCs) [6]. The role of IL-22 is to conduct signal transduction through its receptor IL-22R1 and IL-10R2 together [7]. IL-22 has both pro- and anti-inflammatory effects, suggesting that IL-22 plays an important role in balancing the inflammatory response. For example, studies have shown that IL-22 can promote allergic airway inflammation response in epicutaneously sensitized mice [8] and can aggravate liver fibrosis by enhancing transforming growth factor-β (TGF-β) signaling in hepatic stellate cells (HSCs) [9]. However, IL-22 is critical for intestinal barrier integrity and intestinal immunity [10, 11]. Dextran sodium sulfate (DSS)-mediated colitis can be ameliorated by supplying exogenous delivery of IL-22 to TREM-1-deficient mice [12]. IL-22 treatment can also alleviate liver inflammation in nonalcoholic steatohepatitis (NASH) mice [13]. In addition, IL-22 can dose-dependently induce the expression of multiple S100 family proteins (S100A7, S100A8, and S100A9) [14], especially S100A9, which can promote M2 macrophage polarization [15]. M2 macrophages play a vital role in inhibiting inflammation, particularly in late-stage endotoxemia. These M2 macrophages can release many anti-inflammatory cytokines to induce the host to enter an immunosuppressive state [16].

F-652 is a recombinant fusion protein consisting of two human interleukin-22 (IL-22) molecules linked to an immunoglobulin constant region (IgG2-Fc). This recombinant human IL-22-Fc fusion protein (F-652) is manufactured by Evive Biotechnology (Shanghai) Ltd, and its safety, pharmacokinetics (PK), tolerability, and biomarkers have been evaluated following a single dose in healthy male volunteers in a randomized, double-blind, placebo-controlled study [17]. Recent research about F-652 showed that it could significantly reduce the inflammation and increase the hepatic regeneration in alcohol-associated hepatitis patients compared to the placebo [18]. Therefore, F-652 can effectively treat inflammation-related diseases.

However, the potential role and underlying mechanisms of IL-22 in endotoxemia are not clear yet [19, 20]. Hence, to investigate the possible function of IL-22 in endotoxemia, F-652 was injected into LPS-induced endotoxemia mice. We discovered that F-652 could reduce the release of inflammatory cytokines, but the number of immune cells infiltrated into the lung and the peritoneal cavity was increased. Therefore, we speculated that F-652 could recruit immunosuppressive cells and inhibit the inflammatory response in the LPS-induced endotoxemia model. This speculation is proved in subsequent experiments showing that LPS-induced endotoxemia can be alleviated by supplying exogenous F-652 to promote M2 macrophage polarization.

## Methods

### Animal experiments

Male C57BL/6 mice (8 weeks old) were purchased from Shanghai Model Organisms Center. The care and use of the mice were all approved by the Ethics committee of the Shanghai University of Chinese Traditional Medicine. F-652 is a recombinant fusion protein consisting of two human interleukin-22 (IL-22) molecules linked to an immunoglobulin constant region (IgG2-Fc). This F-652 was provided by Evive Biotechnology (Shanghai) Ltd. In LPS-induced endotoxemia model, sublethal dose (1 mg/kg) or lethal dose (10 mg/kg) LPS (Sigma) and 1 mg/kg F-652 were i.p. injected into mice.

### Isolation of peritoneal macrophages and Bronchoalveolar lavage (BAL) cells

For peritoneal macrophages, 5 ml ice-cold PBS (Gibco) was injected into the peritoneal cavity of mice, and peritoneal lavage fluid was harvested. After centrifugation, peritoneal macrophages were cultured or analyzed by flow cytometry.

For BAL, mice were anesthetized with isoflurane/oxygen inhalational gas (Yuyan). The lungs were washed three times with ice-cold PBS to harvest BAL fluid. After centrifugation, cells and BAL fluid were separated. Cells were counted by hemocytometer; IL-6 concentration was analyzed in BAL fluid by for ELISA.

### Flow cytometry

Single-cell suspensions from peritoneal lavage fluid were stained with a combination of fluorescently conjugated monoclonal antibodies. All antibodies (CD45, CD11b, Ly6G, Ly6C, and F4/80) were purchased from BD Biosciences. For sorting cells (BD FACSAria II), peritoneal macrophages were harvested from 5 mice per one sorting session.

### Histopathology

Lungs were harvested from C57BL/6 mice treated with LPS or LPS + F-652. Then they were fixed in 4% paraformaldehyde at least 24 h, and then embedded in paraffin. 5-μm thickness sections were cut and stained with hematoxylin and eosin (H&E).

### RNA extraction and real-time PCR analysis

The total RNA was extracted with an RNA isolation kit (BioTeke) according to the manufacturer’s instructions, and was reversely transcribed to cDNA (Vazyme). Real-time PCR was performed using SYBR Green (Vazyme). All mRNA genes were normalized to GAPDH. IL-6 forward: TGTTCTCTGGGAAATCGTGGA; IL-6 reverse: TTTCTGCAAGTGCATCATCGT; TNF-α forward: TTCTATGGCCCAGACCCTCA; TNF-α reverse: TTTGCTACGAC.

GTGGGCTAC; Ym 1 forward: CAGGTCTGGCAATTCTTCTGAA; Ym 1 reverse: GTCTTGCTCATGTGTGTAAGTGA; Arg forward: CTCCAAGCCAAAGTCCTTA.

GAG; Arg reverse: AGGAGCTGTCATTAGGGACATC; CCL17 forward: TACCATGAGGTCACTTCAGATGC; CCL17 reverse: GCACTCTCGGCCTACAT.

TGG; S100A9 forward: GGTGGAAGCACAGTTGGCA; S100A9 reverse: GTGTCC.

AGGTCCTCCATGATG; Gapdh forward: AGGCCGGTGCTGAGTATGTC; Gapdh reverse: TGCCTGCTTCACCACCTTCT.

### Enzyme-linked immunosorbent assay (ELISA)

The levels of inflammatory cytokines were detected by ELISA kits (NeoBioscience). The levels of SAA and S100A9 were detected by ELISA kits (Shanghai Enzyme-linked Biotechnology) according to the manufacturer’s instructions.

### Statistical analysis

All results were presented as mean ± SEM from at least three independent biologically replicated experiments, and the log-rank (Mantel-Cox) test was used to analyze survival curves. Data were analyzed using Student’s t-test. *P < 0.05; **P < 0.01; ***P < 0.001.

## Results

### Supplying exogenous F-652 protects mice against LPS-induced endotoxemia

**Fig. 1:**
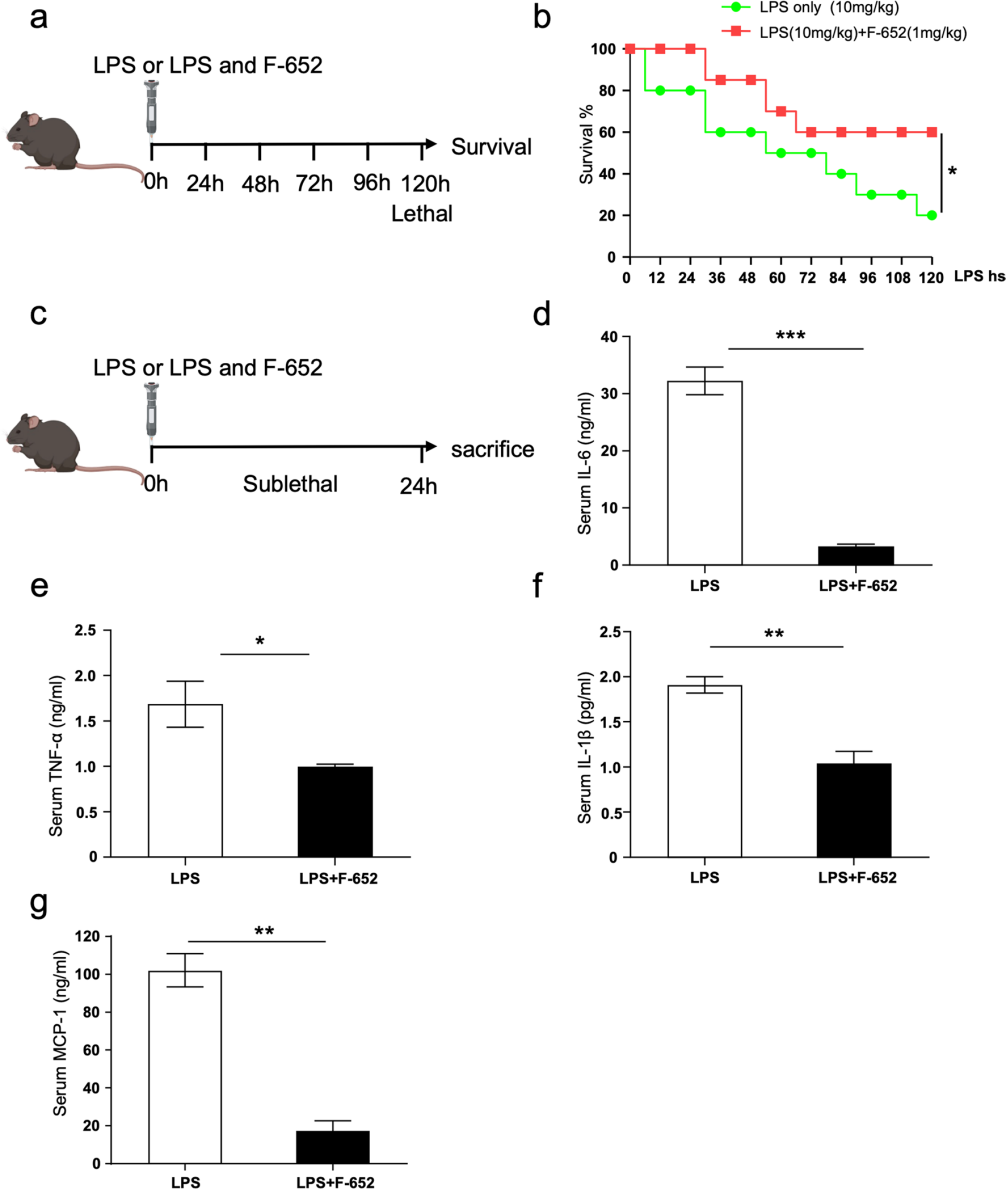
F-652 ameliorates LPS-induced endotoxemia. Mice with F-652 injection show higher survival rate with lower reactive immune responses in LPS endotoxemia model. **a** Schematic diagram showing the mice treated with F-652 strategy in the lethal endotoxemia model. **b** Survival rate (n = 10). **c.** Schematic diagram showing the mice treated with F-652 strategy in the sublethal endotoxemia model. **d–g** The concentrations of pro-inflammatory cytokines in serum (n = 5, or n = 3). The data are shown as means ± SEM. *P < 0.05, **P < 0.01

Previous studies have demonstrated that IL-22 plays an important role in regulating inflammatory responses [10, 19]. Short-chain-fatty acids (SCFA) promote IL-22 production, thereby protecting the intestines from inflammation [10]. In LPS-induced acute liver injury (ALI), pre-treatment with recombinant IL-22 can reduce inflammatory response [19]. To explore the role of IL-22 in the LPS-induced endotoxemia mice model, we treated mice with LPS or LPS plus F-652. The mortality rate of the group with F-652 treatment was significantly lower than that of the group with LPS-treatment only (Fig. 1a, b). In addition, in the low-dose LPS induced nonlethal model, the levels of IL-6, TNF-α, IL-1β, and MCP-1 in serum were significantly lower in LPS + F-652-treated mice than in LPS-treated mice (Fig. 1c–g). These data showed that mice with F-652 injection had a higher survival rate and a less reactive immune response in the LPS endotoxemia model.

### F-652 treatment induced lower cytokines secretion but higher local immune cells recruitment in the LPS-induced endotoxemia model

**Fig. 2:**
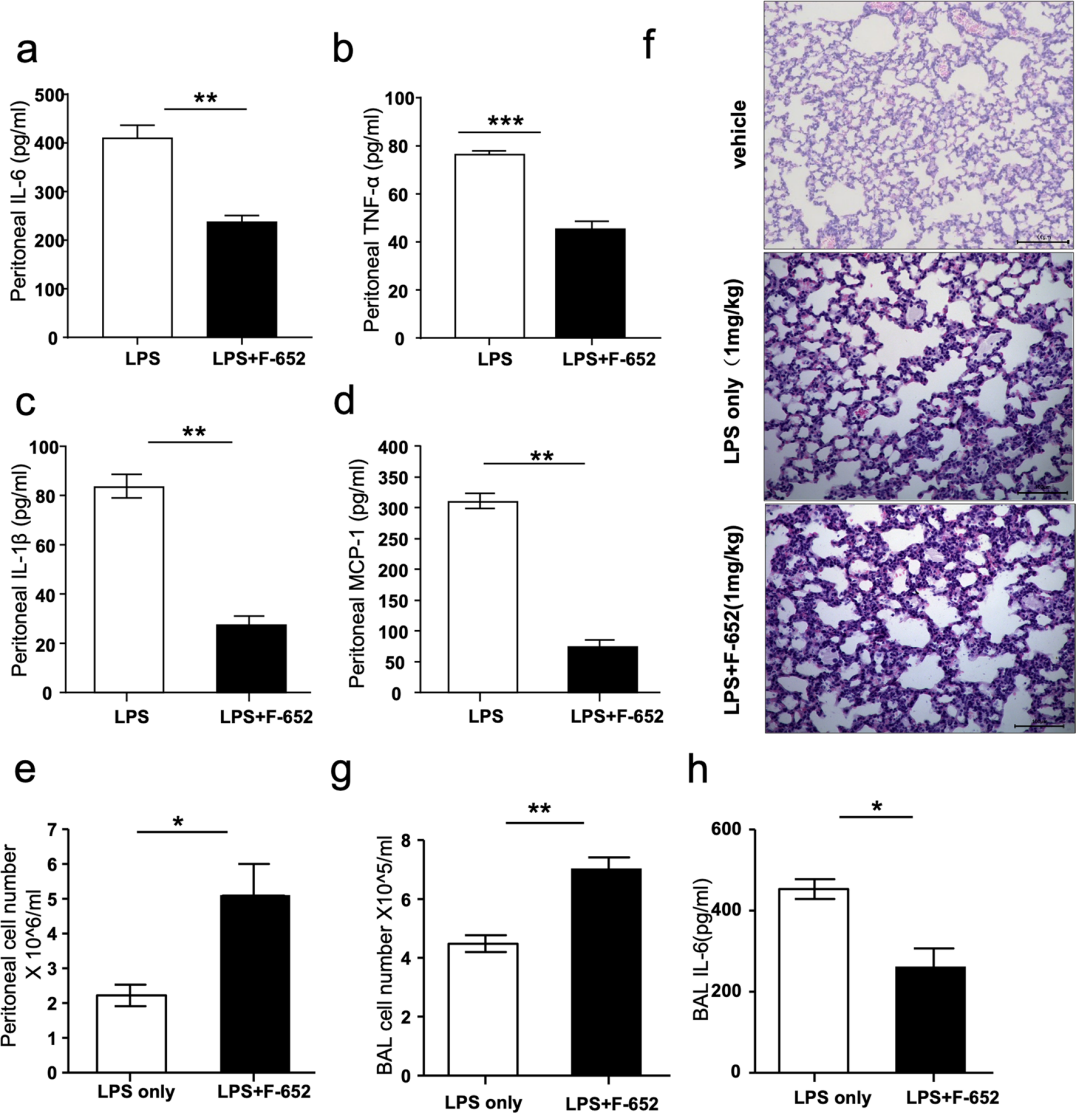
F-652 can increase the cell number in LPS-induced (1 mg/kg) endotoxemia model. **a–d** The concentrations of pro-inflammatory cytokines in peritoneal cavity (LPS: n = 3 or n = 5, LPS + IL-22: n = 4 or n = 5). **e** The cell number of peritoneal lavage fluid (LPS: n = 6, LPS + IL-22: n = 7). **f** Representative images of histopathology of sections of lung tissues that were prepared from LPS-treated and LPS + F-652-treated mice and stained with HE (magnifition 200×). **g** The cell number of bronchoalveolar lavage fluid (n = 4), and **h.** the concentration of IL-6 in BAL (n = 3). The data are shown as means ± SEM. *P < 0.05, **P < 0.01

To further investigate the role of F-652 in the LPS-induced (1 mg/kg) endotoxemia model, we analyzed the local inflammation responses of the mice. Based on the ELISA results, the levels of IL-6, TNF-α, IL-1β, and MCP-1 in peritoneal lavage fluid were significantly lower in the LPS + F-652-treated mice than in the LPS-treated group (Fig. 2a–d). However, the number of peritoneal lavage cells in the LPS + F-652-treated mice was higher (Fig. 2e). A similar result was also observed in lung histopathology and bronchoalveolar lavage analysis. Results suggested that there were more immune cells infiltrated into the lung of the LPS + F-652-treated mice than that of the LPS-treated mice (Fig. 2f), but the level of IL-6 in BAL of the F-652-treated mice was lower (Fig. 2g, h). These data illustrated that, although F-652 increased immune cell infiltration, it actually reduced the release of inflammatory cytokines.

### F-652 increases the immunosuppressive macrophages population

**Fig. 3:**
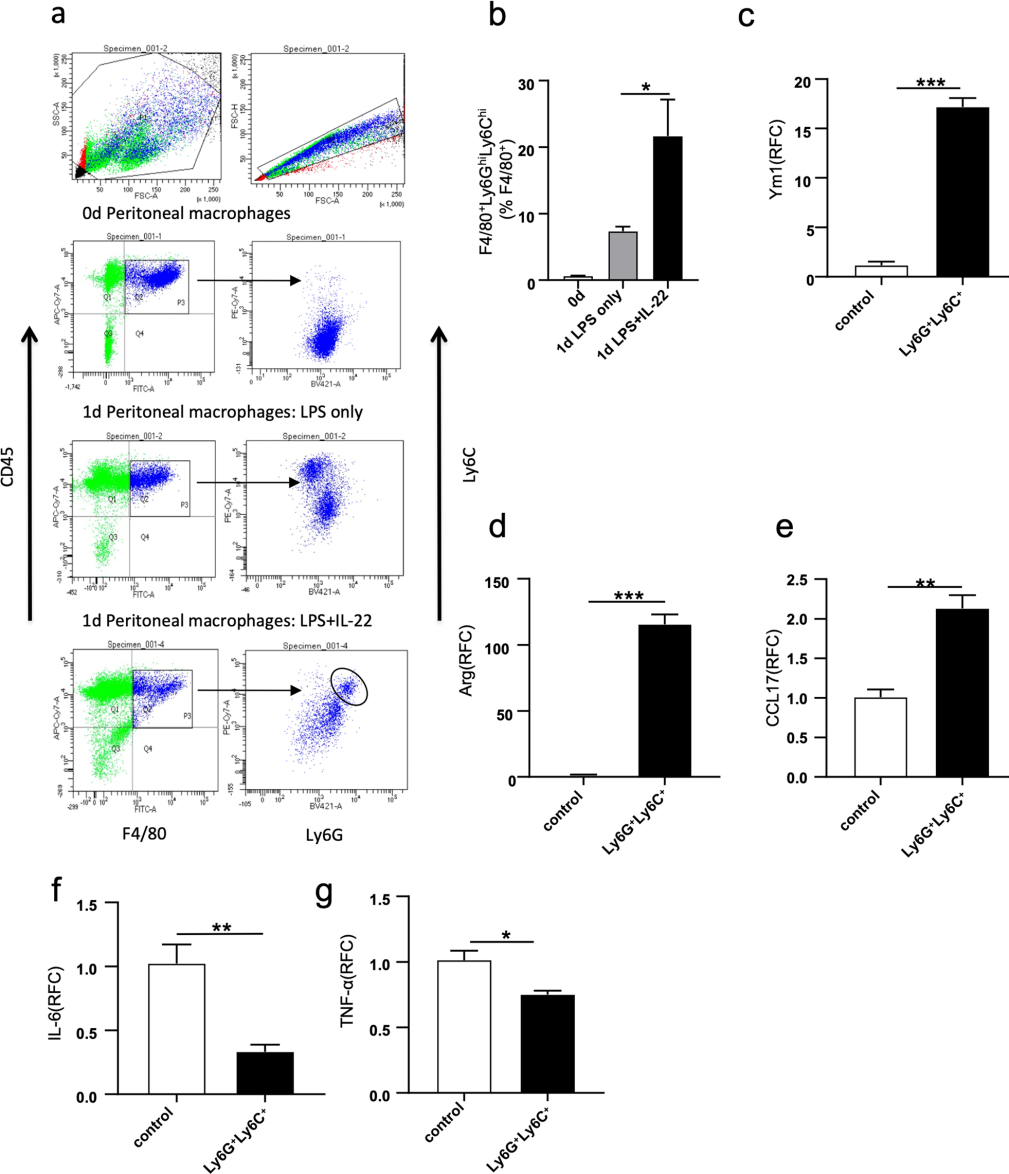
Accumulation of F4/80^+^Ly6G^hi^Ly6C^hi^ cells in peritoneal cavity after addition of F-652 into LPS endotoxemia mice. **a** IL-22 induced the accumulation of F4/80^+^Ly6G^hi^Ly6C^hi^ cells in peritoneal cavity in F-652 treated endotoxemia mice. **b** The statistical quantification of the percentage of the F4/80^+^Ly6G^hi^Ly6C^hi^ M2-like suppressible cells (n = 3). **c–g** The expression of Ym1, Arg, CCL17, IL-6, and TNF-α in F4/80^+^Ly6G^+^Ly6C^+^ cells (control: n = 3, Ly6G^+^Ly6C^+^: n = 4). The data in **b** is analyzed by one-way ANOVA. The data in **c–g** are shown as means ± SEM. *P < 0.05, **P < 0.01

We found that F-652 treatment increased immune cell infiltration but reduced the release of inflammatory cytokines in the LPS-induced endotoxemia model. This result implied that the immune cells recruited by F-652 are immunosuppressive. Since macrophages play an essential role in the inflammatory response of endotoxemia, we examined the types of macrophages in the peritoneal cavity of mice. We found that F4/80^+^Ly6G^hi^Ly6C^hi^ cells in the peritoneal cavity increased after treating the LPS-induced endotoxemia mice with F-652 (Fig. 3a, b). Ly6G^hi^ inflammatory monocytes can promote their polarization into M2-type macrophages in a STAT6-dependent manner [21]. To further analyze the functions of these F4/80^+^Ly6G^hi^Ly6C^hi^ cells, we sorted them and analyzed their expression of the M2 markers. As shown in Fig. 3c–g, compared to control, these cells had significant increases in expressing Ym1, Arg, and CCL17 and had significant decreases in expressing IL-6 and TNF-α. Therefore, F4/80^+^ Ly6G^+^Ly6C^+^ cells from the peritoneal cavity of the F-652-treated mice highly expressed M2 markers, indicating they are the suppressive type of the macrophages.

### S100A9 plays an important role in F4/80^+^Ly6G^+^Ly6C^+^ cells development

**Fig. 4:**
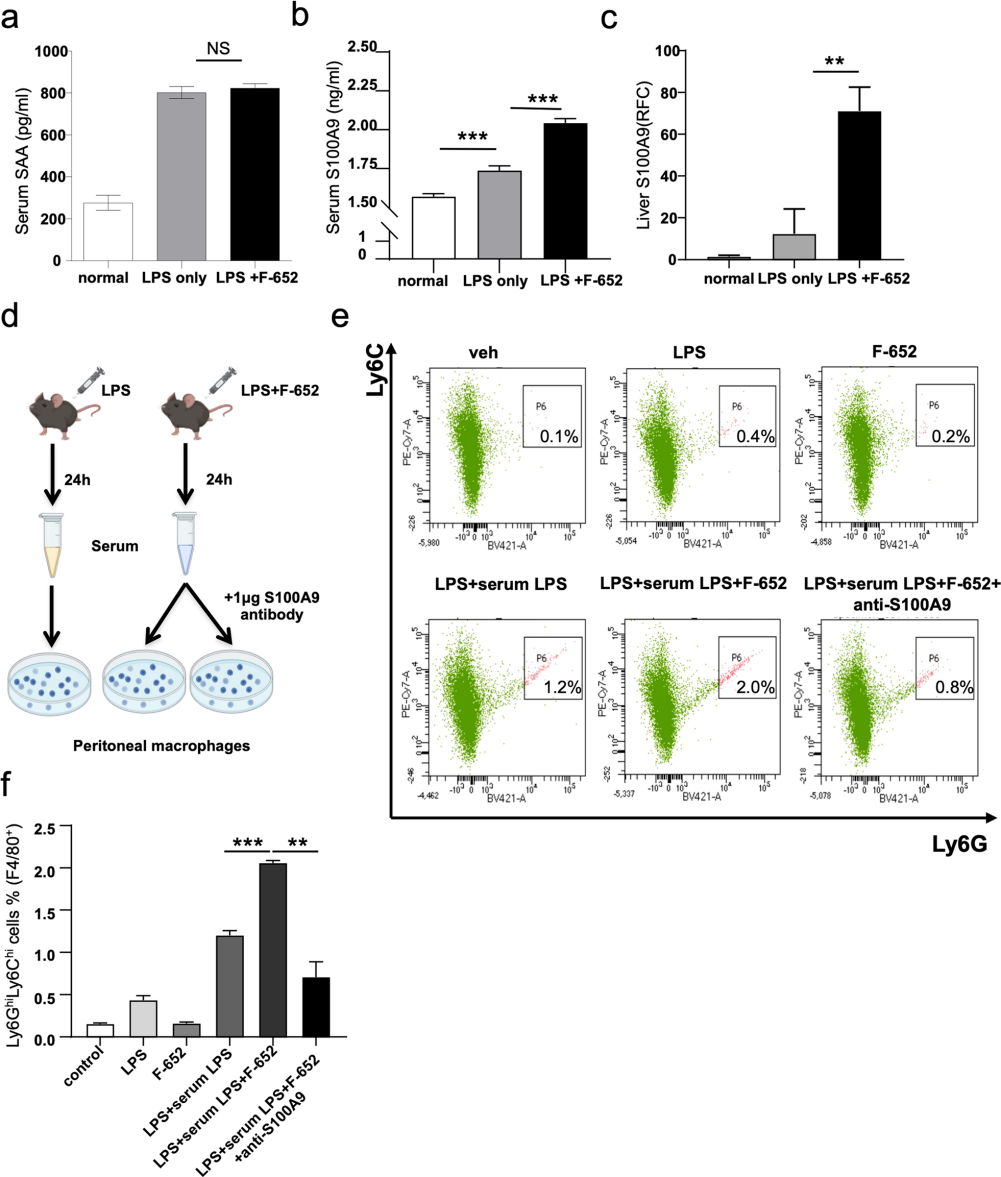
S100A9 is the downstream target of F-652 and plays a pivotal role in developing F4/80^+^Ly6G^hi^Ly6C^hi^ M2-like suppressible cells. **a**, **b** The serum level of SAA (n = 5) and S100A9 (n = 10). **c** The expression of S100A9 in liver (n = 3). **d** Schematic diagram showing the macrophage polarization in vitro. **e** FACS analysis of the percentage of the F4/80^+^Ly6G^hi^Ly6C^hi^ M2-like suppressible cells. **f** The statistical quantification of the percentage of the F4/80^+^Ly6G^hi^Ly6C^hi^ M2-like suppressible cells (n = 3). The data are analyzed by one-way ANOVA. *P < 0.05, **P < 0.01

Because the receptors for IL-22 are restricted to nonhematopoietic cells, and since the systemic inflammation in which IL-22 regulates is dependent on the induction of an acute phase response [22], we suspected that IL-22 might modulate the immune response by regulating the expression of hepatic acute phase proteins (APPs), such as serum amyloid A (SAA) [22]. In addition, IL-22 can dose-dependently induce the expression of multiple S100 family proteins (S100A7, S100A8, and S100A9) [14]. Both SAA and S100A9 play critical roles in inflammatory diseases and can promote the differentiation of M2-type macrophages [15, 23]. Therefore, we examined the serum levels of SAA and S100A9 in the LPS- and the LPS + F-652-treated mice. We found that there are significant differences in the levels of S100A9 but comparative levels of SAA in LPS + F-652-treated mice compared to the LPS group (Fig. 4a, b). Since APPs are mainly from the liver, and because hepatocytes are the main target cell of IL-22 [22], we further detected the expression of S100A9 in the liver. We found that the F-652 treatment induced a significant increase in the expression of S100A9 in the liver (Fig. 4c). To further analyze whether the role of IL-22 in F4/80^+^Ly6G^hi^Ly6C^hi^ cells differentiation is dependent on serum S100A9, we treated peritoneal macrophages with LPS, F-652, or the serum from LPS-treated or LPS + F-652-treated mice with or without S100A9 antibody. As shown in Fig. 4d, the results suggested that treatment with F-652 only has no effects on the induction of Ly6C^hi^ cells. The serum from LPS-treated or LPS + F-652-treated mice induced a significant increase in Ly6C^hi^ cells while blocking S100A9 resulted in a significant decrease in Ly6C^hi^ cells (Fig. 4d–f). Thus, more suppressive Ly6C^hi^ cells in LPS + F-652-treated mice probably because of the high S100A9 levels in the serum.

**Fig. 5:**
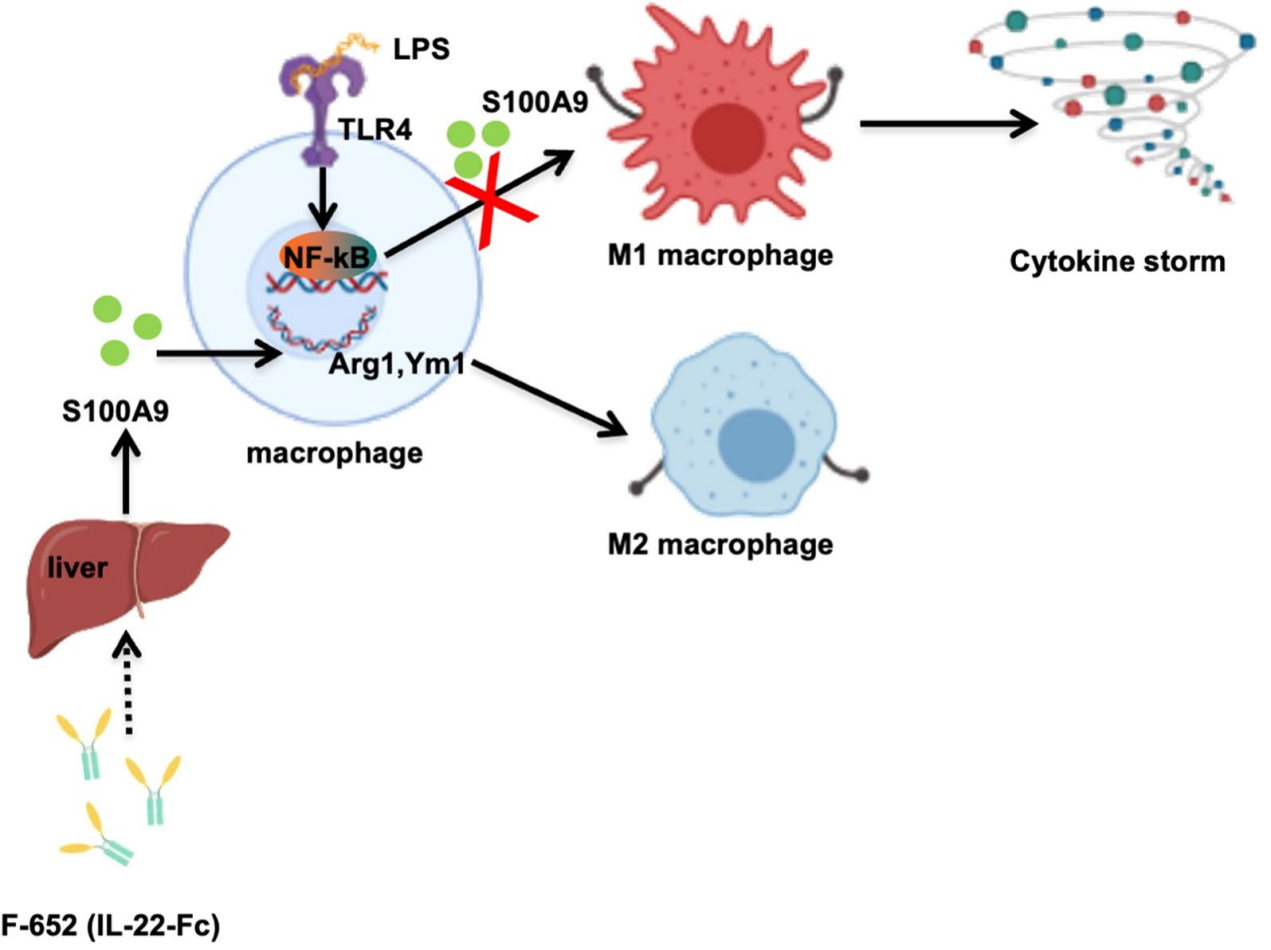
Pivotal function of F-652 in LPS-induced endotoxemia. In the LPS induced endotoxemia model, LPS are rapidly recognized by pathogen recognition receptors (PRRs), such as Toll-like receptor (TLR4), which can initiate highly specific pro-inflammatory signaling pathways, and release a lot of pro-inflammatory cytokines. F-652 can induced the expression of S100A9 in liver, and S100A9 induced M2 macrophages polarization to suppression of excessive inflammation

We summarized our study in Fig. 5. IL-22 (F-652) protects against LPS-induced cytokines storm in the LPS-induced endotoxemia model by inducing the expression of APPs from the liver, like S100A9, and thereby inducing F4/80^+^Ly6G^hi^Ly6C^hi^ M2-like suppressor cells differentiation.

## Discussion

There are many types of lymphocyte subpopulations, such as Th17, Th22, and ILC3s, that could produce IL-22 [24]. Multiple studies have illustrated that IL-22R, one of the IL-22 receptors, is preferentially expressed in epithelial cells, and IL-22 acts mainly on tissue epithelial and stromal cells to induce tissue repair and innate host responses [25]. Numerous reports have shown that IL-22 modulates systemic immunity by acting indirectly on immune cells, and the liver plays an essential role in this process [7]. For example, in bacteria-induced lung infections, IL-22 acts on the liver to induce the expression of C3 and, thereby, modulates immune responses to control the infection [26]. These studies have demonstrated that IL-22 plays important functions in systemic and local immunity by indirectly acting on immune cells.

There are also many controversial studies about the effects of IL-22 in modulating the immune response. IL-22 has been reported to be involved in both pro- and anti-inflammatory effects. For example, Interleukin-22 deficiency alleviates doxorubicin-induced oxidative stress and cardiac injury via the p38 MAPK/macrophage/Fizz3 axis in mice [27]. In another study about the rIL-22-treated ApoE^−/−^ mice, the levels of IL-6 were significantly increased. This increase in IL-6 concentration promoted the maturation of DC and, thereby, induced Th17 cell proliferation and exacerbated inflammation [28]. However, most studies on the anti-inflammatory effects of IL-22 have focused on its ability to maintain the integrity of the intestinal barrier and eliminate bacteria [10, 29].

There were also several studies about the direct effects of IL-22 in modulating immune cells. In 2019, Eun-Young Kim and his colleagues discovered that IL-22 treatment could directly promote the polarization of M2 macrophages [30]. Subsequently, in 2020, Si-BiaoSu et al. found that IL-22 could promote the differentiation of Kupffer cells into the M2-like macrophages [31]. As one of the primary innate immune cells, macrophages play an important role in endotoxemia. However, there are very few studies on the regulation of macrophages by IL-22. Therefore, it remains unclear whether IL-22 plays an important role in endotoxemia and whether it can regulate the polarization of M2 macrophages in the LPS-induced endotoxemia model. In the current study, we adopted a recombinant fusion protein containing human IL-22 called F-652. According to the results of clinical research, F-652 is a safe and well-tolerated drug that demonstrates favorable PK and pharmacodynamic properties [17]. In addition, F-652 can significantly reduce inflammation and increase hepatic regeneration in patients with alcohol-associated hepatitis compared to the placebo [17, 18]. Therefore, F-652 has the potential to treat inflammatory diseases, and we thereby investigated the role and potential mechanisms of F-652 in endotoxemia.

In this study, we found that LPS + F-652-treated mice had improved mortality rates and decreased the production of pro-inflammatory cytokines (IL-6, TNF-α, Mcp-1, and IL-1β) compared to the mice treated with LPS only (Fig. 1). In contrast, the number of immune cells in the lung, peritoneal lavage fluid, and bronchoalveolar lavage fluid was significantly increased in the LPS + F-652-treated mice (Fig. 2). More immune cells with lower cytokine production implied that these immune cells exhibited a suppressive immune response. Since IL-22 could promote the polarization of the M2-like macrophages, we hypothesized that F-652 might promote the polarization of the M2-like macrophages and thus suppress the inflammatory response in the LPS-induced endotoxemia model. Subsequently, our results demonstrated that the addition of F-652 in the LPS-induced endotoxemia mice resulted in more F4/80^+^Ly6G^hi^Ly6C^hi^ cells in the peritoneal cavity, showing a generic signature of M2-type macrophage (Fig. 3). To investigate whether IL-22 directly induces M2 macrophage polarization, we isolated macrophages and treated them with LPS or LPS + F-652 in vitro. Results showed that F-652 treatment did not increase Ly6C^hi^ cells, but the treatment with serum from F-652-treated mice induced macrophages to differentiate into F4/80^+^Ly6G^hi^Ly6C^hi^ cells (Fig. 4d–f). Thus, our results suggest that IL-22 promotes M2 macrophage polarization not by acting directly on macrophages but through hepatic APPs in the serum.

The liver is the main target organ of IL-22. Studies have shown that hepatocytes are the primary target of IL-22, since it can induce hepatic production of acute-phase proteins [32]. IL-22 can affect hepatic gene expression 30 min after injection [22]. By using serum from LPS + F-652-treated mice, Ly6C^hi^ cells were significantly enhanced compared to other groups (Fig. 4e, f). This suggests that APPs in the serum are involved in the induction of Ly6C^hi^ cells. In addition, IL-22 can dose-dependently induce the expression of multiple S100 family proteins (S100A7, S100A8, and S100A9) [14]. Both SAA and S100A9 play important roles in inflammatory diseases and can promote the differentiation of M2-type macrophages [15, 23, 33]. Therefore, we examined the serum levels of SAA and S100A9 in the LPS- and the LPS + F-652-treated mice. We found that there is no significant difference in the level of SAA between LPS- and LPS + F-652-treated mice (Fig. 4a–c). However, the level of S100A9 in LPS + F-652-treated mice was higher than that of the LPS-treated mice (Fig. 4b). Then, we found that S100A9 was significantly increased in the liver of F-652-treated mice (Fig. 4c). More importantly, the blockage of S100A9 decreased the polarization of the F4/80^+^Ly6G^hi^Ly6C^hi^ cells (Fig. 4e, f). Therefore, IL-22 induced more suppressive Ly6Chi cells in the LPS-induced endotoxemia model, probably by inducing S100A9 production in the liver.

In summary, our findings suggest that F-652 (IL-22) protects mice against LPS-induced endotoxemia by promoting M2-like macrophage polarization, and this process may depend on S100A9 (Fig. 5).

## Data Availability

The datasets used and/or analysed during the current study are available from the corresponding author on reasonable request.

## Abbreviations

ALI: Acute liver injury
APPs: Acute phase reaction proteins
DSS: Dextran sodium sulfate
Elisa: Enzyme-linked immunosorbent assay
HSCs: Hepatic stellate cells
H&E: Hematoxylin and eosin
IL-22: Interleukin 22
IL-22BP: IL-22-binding protein
IL-6: Interleukin-6
ILCs: Innate lymphoid cells
IgG2-Fc: Immunoglobulin constant region
LPS: Lipopolysaccharide
NASH: Nonalcoholic steatohepatitis
OECD: Organization for Economic Cooperation and Development
PK: Pharmacokinetics
SAA: Serum amyloid A
SCFA: Short-chain-fatty acids
TNF-α: Tumor necrosis factor α
TGF-β: Transforming growth factor-β
TREM-1: Triggering receptor expressed on myeloid cells-1
RFC: Relative fold change
